# Platelets as Modulators of Cerebral Ischemia/Reperfusion Injury

**DOI:** 10.3389/fimmu.2019.02505

**Published:** 2019-11-01

**Authors:** David Stegner, Vanessa Klaus, Bernhard Nieswandt

**Affiliations:** ^1^Institute of Experimental Biomedicine–Department I, University Hospital Würzburg, Würzburg, Germany; ^2^Rudolf Virchow Center for Experimental Biomedicine, University of Würzburg, Würzburg, Germany

**Keywords:** thrombo-inflammation, ischemic stroke, platelet, glycoprotein Ibα, platelet degranulation

## Abstract

Ischemic stroke is among the leading causes of disability and death worldwide. In acute ischemic stroke, the rapid recanalization of occluded cranial vessels is the primary therapeutic aim. However, experimental data (obtained using mostly the transient middle cerebral artery occlusion model) indicates that progressive stroke can still develop despite successful recanalization, a process termed “reperfusion injury.” Mounting experimental evidence suggests that platelets and T cells contribute to cerebral ischemia/reperfusion injury, and ischemic stroke is increasingly considered a thrombo-inflammatory disease. The interaction of von Willebrand factor and its receptor on the platelet surface, glycoprotein Ib, as well as many activatory platelet receptors and platelet degranulation contribute to secondary infarct growth in this setting. In contrast, interference with GPIIb/IIIa-dependent platelet aggregation and thrombus formation does not improve the outcome of acute brain ischemia but dramatically increases the susceptibility to intracranial hemorrhage. Here, we summarize the current understanding of the mechanisms and the potential translational impact of platelet contributions to cerebral ischemia/reperfusion injury.

## Introduction

Stroke is a leading cause of disability and death worldwide. The majority of strokes are caused by cerebral ischemia, only ~20% of strokes are caused by primary intracerebral hemorrhage (1). The primary therapeutic goal in acute ischemic stroke is the rapid re-establishment of blood flow by thrombolysis or mechanical thrombectomy. The invention of thrombectomy has dramatically increased the rate of successful recanalization up to 80% and improved neurological sequelae, but the *number needed to treat* (NNT) for a good outcome is still high, around 4–6 (2, 3). It is known from experimental stroke models, that infarcts can grow despite successful reperfusion, which is commonly referred to as ischemia/reperfusion injury (I/RI). In humans, there are many possible reasons for unfavorable outcomes after recanalization such as advanced stroke development with low ASPECTS scores already at treatment entry or bleeding complications, but recent clinical trials testing immune-modulatory drugs in acute stroke indicate that infarcts can grow after restoration of cerebral blood flow also in humans (4, 5), although the occurrence of reperfusion injury in the human brain is less clear than in experimental animal models (6). However, the process of further tissue injury upon reconstitution of blood flow is known to apply to different other organ systems such as heart, liver and kidney (7). The middle cerebral artery (MCA) is the most commonly affected blood vessel in human occlusive/ischemic stroke. A broad range of MCAO models has been developed [reviewed in (8)] but no animal model perfectly reflects the disease under study and each model has strengths and limitations trying to reproduce the complex heterogeneous nature of stroke in humans. To study the underlying pathomechanisms of I/RI in the brain, the transient middle cerebral artery occlusion (tMCAO) model of focal cerebral ischemia is widely used, mostly in rodents (9). Advantages and disadvantages of the tMCAO model have been discussed in a recent review (10). In this model, a filament is usually inserted via the internal carotid artery to occlude the MCA for defined time periods, most commonly for 1 h, resulting in complete infarction of the MCA territory. Importantly, infarcts are not fully developed immediately after recanalization but evolve in the reperfusion phase. Numerous experimental studies have established a contribution of platelets and immune cells, in particular T cells, in this cerebral I/RI (11). Therefore, ischemic stroke is now considered a thrombo-inflammatory disease (12). The contribution of neuro-inflammation to cerebral damage following ischemic stroke has been recently reviewed (11, 13, 14), thus, in this review, we focus on the contribution of platelets.

The initial capture of circulating platelets to exposed components of the extracellular matrix (ECM) at sites of vascular injury or inflammation is mediated by the interaction of the glycoprotein (GP) Ib-IX-V receptor complex with von Willebrand factor (vWF) (15). GPIbα-vWF interactions are essential for initial platelet tethering under high shear flow conditions, found e.g., in stenosed arteries, but are only transient and too weak to mediate firm platelet adhesion, but rather decelerate and recruit platelets from the blood stream, which is reflected by the “rolling” of platelets on the vessel wall (16). These “rolling” platelets can interact via their activatory platelet receptors with the corresponding ligands. Platelet activation is mainly triggered through two major signaling pathways, depending on the initial stimulus: signaling via G-protein coupled receptors (GPCRs) (17, 18) or the (hem)*immunoreceptor tyrosine-based activation motif* (ITAM)-bearing receptors. Both signaling pathways culminate in activation of intracellular signaling cascades involving a rise in cytosolic Ca^2+^ concentration, cytoskeletal rearrangements, mobilization of α- and dense granules and subsequent release of secondary platelet agonists and the conformational change of integrin adhesion receptors, most notably αIIbβ3 (GPIIb/IIIa), from a low to a high affinity state, thereby mediating firm platelet adhesion, aggregation and thrombus growth (19).

Efficient blockade of GPIIb/IIIa, leading to abolished platelet aggregation (20, 21), led to intracranial hemorrhage and the treatment did not reduce cerebral infarct sizes in mice (22). Similarly, in a clinical trial, anti-GPIIb/IIIa treatment of patients in acute ischemic stroke was not protective (23). These data exclude platelet aggregation as a critical pathomechanism underlying stroke progression after recanalization and demonstrate that cerebral I/RI cannot simply be explained by secondary thrombotic events in the microcirculation. This does not exclude the appearance of micro-thrombi in the cerebral microvasculature after ischemic stroke, as thrombo-inflammation might be associated with re-thrombosis but does not require these thrombotic events to cause tissue damage.

## GPIbα-vWF Interactions

**Figure 1:**
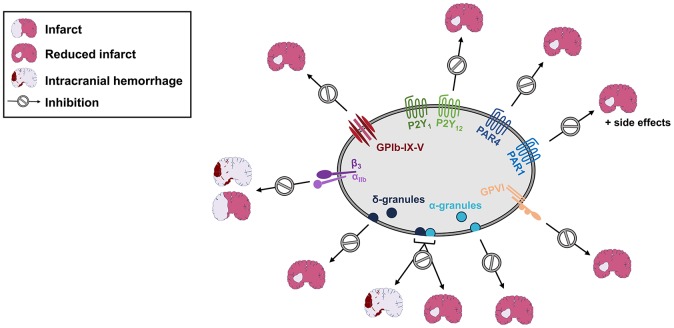
Inhibition of platelet activation or degranulation, but not GPIIb/IIIa blockade results in reduced infarct sizes in experimental stroke. Blockade or genetic deficiency of GPIbα, GPVI, P2Y_1_, P2Y_12_, PAR1, or PAR4 reduces infarct sizes following cerebral ischemia/reperfusion injury. Likewise, lack of either dense (δ) or α-granule secretion improves the outcome following experimental stroke. In contrast, combined loss of dense and α-granule secretion or GPIIb/IIIa-blockade is accompanied by intracranial hemorrhage. See text for details.

GPIbα is part of the GPIb-IX-V receptor complex that is expressed on the platelet surface. The GPIb subunit binds many ligands, including its major ligand vWF, P-selectin, macrophage antigen 1 (Mac-1), and the coagulation factors XI, XII, and thrombin (24). At high shear rates, prevailing in small arterioles or stenosed arteries, initial adherence of platelets to the ECM is mediated by the interaction of GPIbα and vWF (15). Blocking the vWF binding site on GPIbα (using Fab fragments of the p0p/B antibody) has been shown to abolish platelet adhesion to the injured carotid artery wall and protects mice from occlusive thrombus formation *in vivo* (25). Remarkably, treatment of mice with p0p/B Fab lead to reduced infarct sizes, sustained reperfusion and a better neurological outcome after tMCAO, irrespective whether treatment was given 1 h before or after vessel occlusion (see Figure 1) (22, 26). Although mice show a prolonged bleeding time after treatment with the GPIbα blocking Fab, MRI analysis excluded the presence of intracerebral hemorrhages (22). These findings could be reproduced using a transgenic mouse model, where the ectodomain of the human interleukin 4 receptor is expressed instead of the ectodomain of GPIbα (IL4Rα/GPIbα), leading to GPIbα deficiency without the loss of the receptor complex from the platelet surface (27–30). Likewise, vWF-deficient mice that were subjected to the tMCAO model had smaller infarcts and less neurological damage than the respective controls, without showing intracranial bleedings (31). Genetic reconstitution of plasma vWF levels restored the susceptibility of these mice to ischemic stroke (31). Of note, despite only constituting 20% of the total vWF protein (32), platelet vWF alone was sufficient to render mice susceptible to experimental stroke (33). Targeting of GPIbα downstream signaling also appears to be beneficial for the outcome after tMCAO, since deficiency or inhibition of phospholipase (PL) D1 and D2 protected mice from acute ischemic stroke (34–36). Further, GPIbα-blockade reduced cerebral damage in experimental stroke of aged and comorbid animals (e.g., atherosclerotic *Ldlr*^−/−^, diabetic or hypertensive mice) indicating that GPIbα-blockade may be a therapeutic option in the future for patients with accompanying common cardiovascular diseases (37).

Studies performed in stroke patients revealed that genetic alterations in the *GP1bA* gene and increased serum levels of vWF are risk factors for stroke (38, 39), and a predictive factor for long term mortality after acute stroke (40). VWF is a large, multimeric protein (up to 20,000 kDa) synthesized in endothelial cells and megakaryocytes that is highly thrombogenic. In the plasma the enzyme *A disintegrin and metalloprotease with thrombospondin type 1 repeats* 13 (ADAMTS13) cleaves vWF multimers into smaller, less active fragments, thereby reducing vWF activity. Mice lacking ADAMTS13 suffered from severely worsened ischemic brain damage following experimental stroke (41, 42), while intravenous administration of recombinant ADAMTS13 prior to reperfusion reduced infarct size and improved functional outcome after focal cerebral ischemia (41). Autoantibodies against ADAMTS13 as detected in most patients with acquired thrombotic thrombocytopenic purpura (TTP), or mutations in the *ADAMTS13* gene as found in patients with hereditary TTP are associated with an increased risk of thrombotic occlusion of micro vessels in different organs, including the brain (43).

Collectively, these studies argue for the GPIb-vWF axis as an attractive target for stroke therapy. Inhibitory toxins and antibodies or aptamers targeting GPIb-vWF interactions have been designed and are currently tested in preclinical or clinical studies for different applications [reviewed as part of (44)]. Anfibatide, a snake venom-derived GPIb antagonist, led to a dose dependent reduction in infarct sizes without inducing intracranial hemorrhages when tested in mice using the tMCAO model (45) and has successfully completed a phase I clinical study (46). Although the anti-vWF nanobody caplacizumab was initially approved in 2018 as treatment for thrombotic thrombocytopenic purpura (47), it was also shown to be an effective therapy in a stroke model using guinea pigs (48), indicating that it might also be beneficial in stroke therapy. One additional promising feature of strategies targeting the GPIb-vWF axis is that they have been shown to exert thrombolytic activity (48–50). Thus, in addition to dampening thrombo-inflammation (22, 51), also the recurrence of stroke-causing thrombi might be reduced.

## Glycoprotein VI

Glycoprotein (GP) VI is a platelet specific activatory receptor for collagen and fibrin. Upon ligand binding, signals are transduced via an immunoreceptor tyrosine-based activation motif (ITAM) which is contained in the non-covalently associated Fc receptor (FcR)γ-chain dimers that are part of the receptor complex (19, 52). Mice lacking GPVI, either by genetic deficiency of the *Gp6* gene or the FcRγ-chain or by antibody-mediated depletion from the platelet surface, display defective platelet responses to collagen but only a marginally impaired hemostasis, while they are profoundly protected from arterial thrombosis (53, 54) and experimental stroke (see Figure 1) (22). Morphological analysis of the infarcted brain areas and MR imaging could exclude the presence of intracerebral bleedings (22). The spleen tyrosine kinase (Syk) acts downstream of ITAM-coupled receptors and is therefore essential also for GPVI signaling. Upon ligand binding, cross-linking of GPVI brings Fyn and Lyn (two Src family tyrosine kinases) into contact with the FcRγ chain, which starts a tyrosine phosphorylation cascade via Syk, *linker of activated T cells* (LAT) and SLP-76 (SH2 domain containing leukocyte protein of 76 kDa) leading to the activation of most notably phospholipase (PL) C γ2 and phosphoinositide 3-kinases (PI_3_K) (19). Syk not only acts downstream of GPVI but also CLEC-2 and, in human platelets, FcγRIIA. In line with the results from GPVI-deficient animals, Syk-deficient mice or mice that were treated with a Syk-inhibitor, were protected from arterial thrombosis and stroke but displayed only slightly increased bleeding times. Of note, the animals were still protected, when the treatment was given therapeutically, i.e., after induction of the infarction (55). In accordance with the data reported about the loss of ITAM-coupled receptors or signaling molecules, a simultaneous loss of the ITAM inhibitory proteins SLAP and SLAP2 dramatically aggravated neurological damage after tMCAO (56).

The protection from arterial occlusion and stroke by GPVI blockade without markedly increased risk of bleeding makes GPVI a potentially attractive target for the treatment of stroke patients in the clinic. Further, it could be shown that platelets of patients with transient ischemic attack or stroke show an elevated expression of GPVI on their surface when compared to control patients (57). To block GPVI-collagen interaction in the context of thrombosis, the GPVI-Fc fusion protein Revacept was developed and successfully tested in animal models, where it protected mice from cerebral ischemia without inducing intracranial hemorrhages (58). In a first study in healthy humans, all doses were well-tolerated and neither prolonged bleeding time nor thrombocytopenia have been observed. Platelet response to collagen was inhibited in a time and dose-dependent manner, whereas platelet aggregation in response to ADP or TRAP was unaffected (59). The effect of Revacept in patients with carotid artery stenosis, transient ischemic attack (TIA) or stroke is currently tested in a phase II clinical study (NCT 01645306). A second phase II study in patients with coronary artery disease has been initiated and is in the phase of patient recruitment (NCT 03312855). Revacept binds to immobilized collagen and thereby indirectly prevents platelet adhesion and activation at sites of collagen exposure whereas it does not interact with fibrin (60). ACT017 (9O12), a humanized Fab fragment against GPVI, which was designed to directly inhibit GPVI on the platelet surface, was shown to inhibit collagen-induced platelet aggregation *ex vivo* and there were no signs of thrombocytopenia or excessive bleeding (61). In addition to blocking the interaction of GPVI with collagen, ACT017 has been shown to block spreading on fibrinogen of human GPVI transgenic mouse platelets (62) and reduces aggregate formation on fibrin in a model of venous thrombosis (63). A phase II clinical trial investigating the effects of ACT017 in patients with acute ischemic stroke is currently under way (NCT 03803007).

## Protease-activated Receptors (PARs) (Thrombin Receptors)

Thrombin activates platelets through the protease-activated receptors (PARs) 3 and 4 in mice (PAR 1 and 4 in humans), which transduce their signals via G-protein coupled receptors (G_q_ and G_12/13_) thereby leading to platelet activation (64). Receptor expression is not restricted to platelets; PARs are expressed in a variety of tissues and cell types like bone marrow, spleen, gastrointestinal tissues, lung, placenta, thyroid, prostate tissues, and the brain, being involved in various cellular processes (65, 66). Thrombin at high concentrations can enter the brain as a result of increased BBB permeability and acts on PARs that are expressed in the central nervous system (on both, glial cells and neurons). Focal ischemia induces the expression of PAR-1 and PAR-3 on microglia and enhances PAR-4 labeling in the penumbra (67). Since PAR-1 is not expressed in mouse platelets, for initial experiments studying the role of platelet PAR-1, other model systems have been used. In preclinical models using cynomolgus monkeys, selective PAR-1 blockade led to potent inhibition of thrombin-induced platelet aggregation without impairing primary hemostatic function (68). A phase III clinical trial (NCT 00526474) showed that the PAR-1 inhibitor vorapaxar is beneficial in the secondary prevention of cardiovascular death or ischemic events, but increases the risk of bleedings in patients with a stroke history (69–71). Vorapaxar is approved for clinical use but its use is limited by a substantial bleeding risk (72). Another PAR-1 inhibitor, atopaxar has been shown to have a good safety profile in terms of bleeding while reducing the number of adverse ischemic events, but was not developed further because it had not tolerable side effects on heart and liver (73).

PAR4 is the only PAR member that is expressed on both, human and murine platelets, and when *Par-4*^−/−^ mice were submitted to the tMCAO model, they displayed markedly reduced infarct sizes, less neurologic impairment and reduced BBB breakdown and cerebral edema formation (see Figure 1) (74). Since PAR-4 in humans is responsive at higher thrombin concentrations, it is hypothesized that initial platelet responses to low thrombin concentration via PAR-1 are important for hemostasis and later stages of platelet activation by thrombin are important for occlusive thrombus formation and more dependent on PAR-4. Targeting PAR-4 while maintaining PAR-1 function might be more selective in preventing thrombotic occlusion while maintaining hemostasis (75). A selective PAR-4 inhibitor (BMS-986120) was identified and tested in a monkey model of occlusive arterial thrombosis, where it showed highly efficacious antithrombotic activity, and although treated animals had a slightly increased risk for bleeding, this was still markedly reduced compared to clopidogrel (same study), abciximab or cangrelor treated animals (75, 76). BMS-986120 was investigated in phase I clinical trials (NCT02208882 and NCT02439190), where a reduction in *ex vivo* platelet activation, aggregation and thrombus formation could be observed without causing an increase in coagulation times or serious adverse events (77). For further clinical development, BMS-986120 is compared with standard therapy in a phase II study of stroke recurrence (NCT02671461) (78). Given the high abundance of PARs in various tissues and cell types and the regulation of the expression in pathological situations like ischemic stroke, targeting these receptors might always have an influence on other cell types either promoting the beneficial effect or, on the other hand, leading to undesired side effects.

## Adenosine Diphosphate (ADP) Receptors

ADP can activate platelets via three purinergic receptors, P2Y_1_, P2Y_12_, and P2X_1_. P2Y_1_ and P2Y_12_ signal via G-protein coupled receptors (G_α*q*_ and G_α*i*_, respectively), and are important for platelet shape change, aggregation, thromboxane A_2_ generation, procoagulant activity, thrombus formation and signal potentiation. P2X_1_ is an ion channel, that causes Ca^2+^ influx upon activation but has negligible role for platelet plug formation (79). Both, the P2Y_1_ and the P2Y_12_ receptor would be potential targets for the treatment of platelet associated diseases. However, P2Y_1_ is widely expressed, which increases the risk of side effects, whereas P2Y_12_ expression is restricted to the platelet surface and the brain, which makes it a more specific target (79). P2Y_12_ inhibitors can be divided into thienopyridines (like clopidogrel and prasugrel) and non-thienopyridines (like ticagrelor and cangrelor). Thienopyridines require metabolic activation and irreversibly inhibit the P2Y_12_ receptor, whereas non-thienopyridines are already active and lead to a reversible receptor inhibition. Treatment of mice with ticagrelor inhibited ADP induced platelet aggregation and reduced infarct sizes while improving neurological function after tMCAO to a greater extent than acetylsalicylic acid. Further, reperfusion of the infarcted brain area was better as increased cerebral blood can be measured in ticagrelor treated mice, compared to vehicle or acetylsalicylic acid (aspirin®) treatment (80). Likewise, P2Y_12_-deficient mice displayed a slightly improved neurological outcome following 45 min tMCAO as compared to wild-type control animals, albeit the authors of that study ascribed the effect to P2Y_12_ in glia cells (81).

In line with the data acquired for ticagrelor in mice, in a non-human primate model of photochemically induced thrombotic ischemic stroke, monkeys that received daily treatments with prasugrel had significantly smaller infarcts and a better neurological function without suffering from an increased risk of hemorrhage (82). In clinical trials assessing the risk of stroke recurrence subsequent to an initial thrombotic event, dual antiplatelet therapy (DAPT) comprising acetylsalicylic acid and clopidogrel was more efficient in reducing the risk of recurrent events in the acute treatment of non-embolic TIA or ischemic stroke (83, 84), but DAPT was not beneficial in the long-term treatment of patients [reviewed in (85)]. In another study, comparing ticagrelor with aspirin®, ticagrelor was not found to be superior to aspirin® in reducing the occurrence of stroke, myocardial infarction or death within the 90 days observation period (83). However, in a study with patients suffering from atherosclerosis, clopidogrel, and ticlopidine could provide a better protection from stroke, myocardial infarction or vascular death when compared to the aspirin® treated cohort (86).

## Glycoprotein-IIb/IIIa-Receptor

Platelet GPIIb/IIIa (integrin αIIbβ3) is the most abundant integrin on the platelet surface and mediates platelet adhesion on the ECM by binding to its main ligand fibrinogen as well as fibronectin and vitronectin. GPIIb/IIIa is essential for platelet aggregation and thrombus formation by bridging adjacent platelets via fibrinogen or, at high shear rates, vWF (24). Given this important role, several GPIIb/IIIa inhibitors have been developed to use its blockade in the prevention of ischemic cardiovascular diseases. There are three approved GPIIb/IIIa inhibitors, abciximab (a recombinant Fab fragment of a monoclonal anti-GPIIb/IIIa antibody), eptifibatide (small peptide), and tirofiban (small non-peptide compound) (87). Several randomized controlled trials could show that the currently available GPIIb/IIIa inhibitors are safe and efficiently prevent complications in perioperative periods, which is why they are recommended as routine emergency treatment for thrombotic complications in ischemic heart disease (88, 89). In the setting of ischemic stroke, however, blocking GPIIb/IIIa in an experimental stroke model markedly increased the risk of intracerebral hemorrhage and mortality and surviving animals still showed infarct volumes that were in the range of control treated mice. Decreasing the degree of receptor blockade improved survival and reduced bleeding complications, but had no impact on infarct size or neurological symptoms (see Figure 1) (22). Notably, these data also largely exclude thrombus formation (e.g., microthrombosis in cerebral vessels) as a central mechanism underlying thrombo-inflammation and infarct progression in the context of ischemic stroke.

Many clinical trials have been conducted assessing the use of anti-GPIIb/IIIa inhibitors in the treatment of acute ischemic stroke or the prevention of stroke [reviewed for tirofiban in (89)]. Although several studies verified the safety profile of tirofiban, results are highly variable concerning beneficial effects of anti-GPIIb/IIIa treatment for functional outcome and mortality. In the setting of rescue stenting in the acute treatment of ischemic stroke, stent patency correlated with tirofiban administration without increasing intracranial hemorrhage or mortality (89, 90). In other studies, however, GPIIb/IIIa inhibitors have been shown to induce a significant increase in fatal intracerebral hemorrhage as reported for tirofiban (91) and abciximab (23) and must therefore be used with caution.

## Platelet Granule Secretion

Platelets contain three major types of granules, namely α-granules, dense granules and lysosomes, which secrete their content upon platelet activation. α-granules account for roughly 10% of the total platelet mass being the most abundant granule type (50–80 granules/mouse platelet) (92). They contain more than 300 different proteins that play roles in diverse processes such as coagulation, platelet adhesion, hemostasis, wound healing, angiogenesis, host defense and tumor growth (92). Dense granules are the second most abundant granule type (5–6 granules/mouse platelet) and their name results from a characteristic electron-dense spot seen in electron microscopic analysis due to high concentrations of Ca^2+^, Mg^2+^, ADP, ATP, and serotonin (93). All of these molecules act as second wave mediators and promote the activation of platelets. Lysosomes contain various proteolytic enzymes like proteases, glycosidases, and cationic proteins with bactericidal activity proteins (94).

### Dense Granule Secretion

Platelet activation results in the release of dense granule content, such as ADP, ATP, serotonin, and calcium, amplifying the activation response and promoting thrombus formation (92, 95). Mice lacking functional Munc13-4 (*Unc13d*^*Jinx*^, *Unc13d*^−/−^) display abolished platelet dense granule secretion resulting in defective hemostasis and impaired experimental thrombus formation (96–98). Following 1 h tMCAO, *Unc13d*^−/−^ mice had smaller infarcts and a better neurological outcome after 24 h compared to control mice (see Figure 1), which was not accompanied by intra-cranial hemorrhages, indicating that platelet dense granule secretion is not required to preserve vascular integrity in the setting of acute cerebral ischemia (98).

Serotonin (5-hydroxytrytamine, 5-HT) is an important neurotransmitter that has key functions within the brain and in the periphery. Apart from the brain, 5-HT is synthetized by tryptophan hydroxylase 1 (TPH1) by the enterochromaffin cells of the gut (99). Platelets are the major pool for peripheral serotonin, despite being unable to synthesize it; however, they take serotonin up from the plasma and store it in their dense granules (100). *5Htt*^−/−^ mice, which lack the serotonin transporter, cannot store serotonin in the platelet dense granules and display defective hemostasis and are protected in models of arterial thrombosis (101). In experimental stroke, however, no differences were observed between *5Htt*^−/−^ and control mice (101), indicating that abolished serotonin secretion is not responsible for the reduced cerebral damage observed in *Unc13d*^−/−^ mice.

Given the well-established role of ADP/ATP in secondary platelet activation (102), it seems more likely that the reduced infarct sizes following brain I/RI in *Unc13d*^−/−^ mice results from the decreased platelet activation due to missing ADP/ATP secretion in these animals. In line with this, ticagrelor-treated mice displayed reduced infarct sizes and better neurological outcome following tMCAO (80). Consequently, the primary contribution of platelet dense granule secretion to cerebral ischemia/reperfusion damage appears to be the amplification of platelet activation.

### Alpha Granule Secretion

Platelet α-granules contain more than 300 different proteins involved not only in platelet adhesion but also inflammation, angiogenesis and wound healing (92). The gray platelet syndrome (GPS) is a rare, autosomal-recessive platelet disorder characterized by thrombocytopenia, large platelets lacking α-granules, and variable bleeding symptoms (103, 104). GPS has been linked to mutations in the neurobeachin-like 2 gene (*NBEAL2*) (105–107) and *Nbeal2*^−/−^ mice mimic the hallmarks of GPS (108–110). They were protected in models of arteriolar thrombosis and ischemic stroke (see Figure 1) and displayed prolonged tail bleeding times (108). Of note, *Nbeal2*^−/−^ mice did not display signs of spontaneous bleeding, nor was intracranial hemorrhage within 24 h after cerebral ischemia observed (108). We are not aware of any epidemiological studies that assessed the occurrence of ischemic stroke in GPS patients compared to the overall population. However, the results with *Nbeal2*^−/−^ mice in experimental stroke indicate that α-granule proteins contribute to cerebral damage, but are not required for vascular integrity following I/RI of the brain.

Interestingly, *Unc13d*^−/−^*/Nbeal2*^−/−^ double KO mice, which are unable to secrete their granule content showed an impaired hemostatic response in the ischemic brain following tMCAO, causing increased intracranial hemorrhage and mortality (111). Platelet transfer experiments confirmed that the platelet granule content is required to prevent intracranial hemorrhage in these mice (111). Of note, *Unc13d*^−/−^*/Nbeal2*^−/−^ mice did not display a defective blood-brain barrier, nor did these mice bleed in experimental models of skin or lung inflammation, indicating that platelet granule content is of particular importance to maintain the integrity of the cerebral vasculature in the course of I/RI (111). The fact that *Unc13d*^−/−^ platelets display up to 60% reduced α-granule secretion at threshold agonist concentrations (98) indicates that only a small amount of α-granule-derived mediators is sufficient to maintain vascular integrity during ischemic stroke. This is supported by studies on thrombocytopenic mice revealing that 5% of normal platelet counts were still sufficient to prevent intra-cranial bleedings in experimental models of focal cerebral ischemia, while only severely thrombocytopenic mice (peripheral platelet counts of <2.5% of control) suffered from intracranial hemorrhages (22, 112, 113). Interestingly, a small fraction of *Unc13d*^−/−^*/Nbeal2*^−/−^ mice did not display intracranial hemorrhages and these animals survived and had smaller infarcts as compared to controls (111).

The plethora of platelet α-granule proteins and the fact that the majority of them can also be released by other cells, makes it challenging to identify the key components that contribute to infarct progression following cerebral ischemia.

## Conclusions and Perspectives

The heterogeneity of stroke patients makes the development of safe antiplatelet treatments challenging. Nevertheless, experimental stroke studies suggest that targeting activatory platelet receptors might be a feasible strategy to reduce thrombo-inflammatory infarct progression following ischemic stroke. Due to the fact that the expression of GPVI and GPIbα is limited to platelets we consider these two receptors particularly interesting in this aspect, as side-effects of their inhibition on other tissues are unlikely. In particular, targeting the GPIbα-vWF interaction could be a very attractive approach to inhibit pathogenic platelet functions in acute ischemic stroke. According to this hypothesis, blocking GPIbα would prevent platelet adhesion and therefore the first step of platelet recruitment/activation in the reperfused cerebral microcirculation. Moreover, GPIbα-blockade also reduces the infiltration of immune cells into the infarcted area and thereby inflammation (51) while not inducing increased intracerebral hemorrhages, at least in the setting of experimental focal ischemia (22). In contrast, GPIIb/IIIa blockade is clearly a less favorable option as it is ineffective in reducing thrombo-inflammation and a full receptor blockade causes bleeding complications.

In the future, it will be of interest to investigate whether thrombo-inflammatory mechanisms already contribute to cerebral damage in the ischemic phase and whether targeting them before recanalization would further reduce infarct progression.

Likewise, treatment strategies that stimulate inhibitory signaling pathways in platelets could be considered in the context of ischemic stroke. Indeed, cilostazol an inhibitor of phosphodiesterase-3 (PDE3), which dampens platelet activity due to sustained levels of cyclic nucleotide monophosphate (cAMP and cGMP), has been shown to reduce recurrent strokes (114, 115). However, data from experimental studies argues that the protective effect of PDE3-inhibitors might be independent of platelet inhibition (116).

## Author Contributions

DS and VK wrote the manuscript. BN conceptualized and edited the manuscript.

### Conflict of Interest

The authors declare that the research was conducted in the absence of any commercial or financial relationships that could be construed as a potential conflict of interest. The handling editor declared a past collaboration with one of the authors, BN.
